# Feeling the beat: a smart hand exoskeleton for learning to play musical instruments

**DOI:** 10.3389/frobt.2023.1212768

**Published:** 2023-06-29

**Authors:** Maohua Lin, Rudy Paul, Moaed Abd, James Jones, Darryl Dieujuste, Harvey Chim, Erik D. Engeberg

**Affiliations:** ^1^ Department of Ocean and Mechanical Engineering, Florida Atlantic University, Boca Raton, FL, United States; ^2^ Department of Mechanical Engineering, Boise State University, Boise, ID, United States; ^3^ Division of Plastic and Reconstructive Surgery, University of Florida College of Medicine, Gainesville, FL, United States; ^4^ Center for Complex Systems and Brain Science, Florida Atlantic University, Boca Raton, FL, United States

**Keywords:** soft robot, exoskeleton, sensor array, hand, artificial intelligence, 3D print

## Abstract

Individuals who have suffered neurotrauma like a stroke or brachial plexus injury often experience reduced limb functionality. Soft robotic exoskeletons have been successful in assisting rehabilitative treatment and improving activities of daily life but restoring dexterity for tasks such as playing musical instruments has proven challenging. This research presents a soft robotic hand exoskeleton coupled with machine learning algorithms to aid in relearning how to play the piano by ‘feeling’ the difference between correct and incorrect versions of the same song. The exoskeleton features piezoresistive sensor arrays with 16 taxels integrated into each fingertip. The hand exoskeleton was created as a single unit, with polyvinyl acid (PVA) used as a stent and later dissolved to construct the internal pressure chambers for the five individually actuated digits. Ten variations of a song were produced, one that was correct and nine containing rhythmic errors. To classify these song variations, Random Forest (RF), K-Nearest Neighbor (KNN), and Artificial Neural Network (ANN) algorithms were trained with data from the 80 taxels combined from the tactile sensors in the fingertips. Feeling the differences between correct and incorrect versions of the song was done with the exoskeleton independently and while the exoskeleton was worn by a person. Results demonstrated that the ANN algorithm had the highest classification accuracy of 97.13% ± 2.00% with the human subject and 94.60% ± 1.26% without. These findings highlight the potential of the smart exoskeleton to aid disabled individuals in relearning dexterous tasks like playing musical instruments.

## 1 Introduction

Patients with neuromuscular disorders commonly face challenges when it comes to engaging in everyday activities. For instance, after a stroke, their ability to carry out daily tasks can be affected due to decreased coordination and strength in one or both of their upper limbs (Lai et al., 2019). As a result, they may experience asymmetric function caused by unilateral hand weakness (Patel and Lodha, 2019). Moreover, spasticity can develop over time and affect their ability to perform personal hygiene tasks, leading to further deterioration of the affected limb’s function (Kerr et al., 2020). This pattern of disability is alsoevident in conditions like cerebral palsy (Gordon et al., 2013). These problems have spurred the development of robotic devices to enhance the abilities of patients recovering from these debilitating disorders (Lum et al., 2012; Polotto et al., 2012; Squeri et al., 2013; Aubin et al., 2014).

Exoskeletons are a relatively new solution for addressing support and enhancing user movements (Tran et al., 2021). Traditionally, exoskeleton systems have consisted of inflexible mechanical structures that are often powered by electric motors (Herder and Antonides, 2006; Rahman et al., 2006; Rahman et al., 2015; Gopura et al., 2016). These systems are precise and offer numerous control techniques (Gopura and Kiguchi, 2008; Kiguchi and Hayashi, 2012; Li et al., 2013; Ambrosini et al., 2014; Jarrassé et al., 2014; Leonardis et al., 2015; Lambelet et al., 2017). They have proven especially beneficial in stable scenarios such as when attached to wheelchairs or if used as standing aids in physical therapy (Iwamuro et al., 2008). Nonetheless, the rigid nature of such assistive devices can create problems of their own. Properly distributing force for user safety and comfort can be challenging in some cases (Mohamaddan and Komeda, 2010; Pons, 2010; Rahman et al., 2015). Although various approaches have been suggested to address this issue, it remains an active area of research (Jarrassé and Morel, 2011).

Surgical interventions to restore upper limb functionality operate in a manner akin to inflexible exoskeletons. When dealing with upper limb spasticity, surgical choices may involve the fractional elongation and release of tendons or targeted neurotomies to reduce the debilitating effects of spasticity. In severe cases, bone surgery like wrist fusion may enhance joint alignment, which could facilitate residual hand function, but this comes at the cost of sacrificing the range of motion in the affected joint. In some neurological conditions, such as brachial plexus injuries, surgery is not able to restore full motor and sensory function equivalent to the uninjured upper extremity. In total brachial plexus injuries, surgery is targeted at restoring the key movements of elbow flexion and shoulder abduction or external rotation—restoration of sensation and motor function in the hand is not possible at that point. Exoskeletons may, therefore, have a complementary role to surgery in managing these patients.

Supportive exoskeletons have played a vital role in aiding patients to manage and recuperate from neurotrauma. Cable and spring-based mechanical exoskeletons have proven to be beneficial in rehabilitation, but their size and complexity often render them impractical in meeting patients’ needs (Arata et al., 2013; In et al., 2015; Nycz et al., 2015; Kang et al., 2016; Nycz et al., 2016; Yang et al., 2016; Jarrett and McDaid, 2017; Li et al., 2018). The fabrication and maintenance of these systems are challenging due to the requirement for custom parts to accommodate the unique anatomy of each patient (Hussain et al., 2016; Kang et al., 2016). Furthermore, the structures used are not ergonomic, and rigid exoskeletons tend to become excessively bulky.

The adoption of soft pneumatic actuators has revolutionized the development of exoskeletons that meet patients’ requirements for lightweight, pliable, and practical support (Andrikopoulos et al., 2015; Al-Fahaam et al., 2016; Haghshenas-Jaryani et al., 2016; Al-Fahaam et al., 2018; Cappello et al., 2018; Li et al., 2020; Lin et al., 2020; Abd et al., 2021). However, the flexibility of these actuators creates a challenge that necessitates the use of flexible sensor technology capable of accommodating and compensating for significant deformation (Yeo et al., 2016). By implementing effective and adaptable sensor techniques, this challenge can be overcome (Haghshenas-Jaryani et al., 2016; Abd et al., 2021).

Relearning tasks involves the restoration and retraining of specific movements or skills. Soft robotic exoskeletons, utilizing the properties of flexible materials and sensors, provide gentle support and assistance to individuals in relearning and regaining their motor abilities (Polygerinos et al., 2015). By monitoring and responding to users’ movements, soft robotic exoskeletons can offer real-time feedback and adjustments, making it easier for patients to grasp the correct movement techniques (Deimel and Brock, 2016). Playing the piano requires complex and highly skilled movements. For individuals who have lost the ability to play due to neurotrauma, soft robotic exoskeletons can serve as powerful assistive tools (Hoang et al., 2021). The flexibility of soft materials and sensors enables the exoskeletons to adapt to the shape and motion of the hand, providing precise force and guidance to aid patients in recovering the fine finger movements required for piano playing (Takahashi et al., 2020). However, achieving precise force control and adaptability requires the development of highly intelligent algorithms to address motion planning issues (Wang and Chortos, 2022).

**FIGURE 1:**
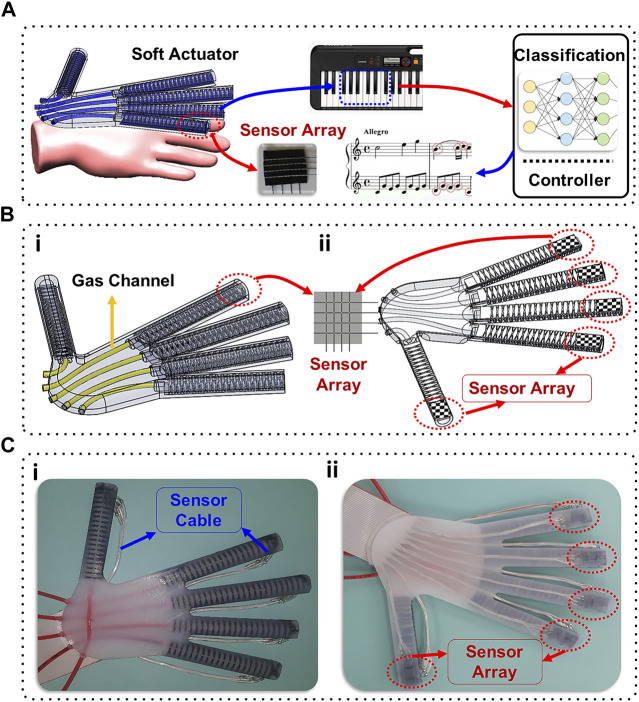
**(A)** Soft actuator with sensor arrays; **(B)** CAD model for the new sensorized soft hand exoskeleton (i) top view, (ii) bottom view; **(C)** The new soft hand exoskeleton (i) top view, (ii) bottom view.

The objective of this study is to introduce a smart assistive hand exoskeleton that comprises five soft pneumatic actuators, each fitted with a 16-taxel flexible sensor at the fingertip (Figures 1A–C). The design and fabrication process of the hand exoskeleton is novel and could be customized to unique anatomy of different patients. This completely soft design will improve user comfort and result in a lightweight convenient exoskeleton. Furthermore, the fabrication is significantly simpler than most designs as all the actuators and sensors are combined into a single molding process. As a demonstration of the exoskeleton’s capabilities to serve as an intelligent assistant, it was employed to ‘feel’ the difference between correct and incorrect versions of a song played on the piano. The implementation of sensing systems within soft exoskeletons has been done less frequently and in this case the sensors are utilized to a much greater extent. Experiments were conducted using the exoskeleton independently and while worn by a human subject to show that one of the myriad possibilities of this new device could be to aid relearning how to play the piano after neurotrauma. The fingertip tactile sensor signals were employed to train three different machine learning algorithms: RF, KNN, and ANN. The accuracy of these algorithms was compared to classify the correct and incorrect song variations with and without the human subject.

## 2 Experimental methods

A soft robotic exoskeleton for the hand was designed to offer active flexion and passive extension assistance to all five digits. The exoskeleton was constructed using Dragon Skin-30 material, which provides passive freedom for transverse movements along the palmar plane for each finger. Additionally, flexible sensor arrays comprising sixteen taxels were incorporated into each fingertip to enable pattern recognition of piano-playing performance.

### 2.1 Sixteen-channel flexible tactile sensor fabrication

The flexible tactile sensor array employs two primary piezoresistive elements: velostat and stainless-steel thread. Velostat is a composite film made from polyethylene and carbon black. The velostat changed conductivity when pressure was applied, so also did the stainless-steel thread exhibit increased conductivity upon the application of force. The application of pressure to the velostat caused the distance between the carbon black particles to decrease and increased their contact points, leading to changes in conductivity across the affected area of the film (Dzedzickis et al., 2020). Similarly, applying force to the stainless-steel thread increased contact between the thread and the film and within the thread, leading to enhanced conductivity (Choudhry et al., 2022). By arranging the threads in a grid pattern that is connected to the conductive film, it is possible to create a sensor array that measures the pressure distribution across the grid’s surface.

The sensor was created by assembling seven layers, which included an outer layer, an adhesive layer, longitudinal wires, a conductive layer, transverse wires, another adhesive layer, and a second outer layer. Plastic wrap was used for the outer layers (MRP Corp, Philadelphia, PA), while the adhesive layer was made of 3 M double-sided adhesive (3 M™ Adhesive Transfer Tape 468MP, United States). The wires were made of a stainless-steel yarn (Stainless Thin Conductive Thread - 2 ply, Adafruit Industries LLC), and the conductive layer was made of velostat, a pressure-sensitive and conductive sheet (Velostat 1361, Adafruit Industries LLC). The assembly process involved cutting rectangles out of the velostat to match the sensor’s size and cutting the plastic wrap and adhesive into a rectangle of 2 cm × 1.5 cm. To aid the assembly, wires were placed in a 3D-printed holster to maintain an even spacing of 0.2 cm apart for the longitudinal wires and 0.3 cm apart for the transverse wires. The wires were then lightly pressed onto one of the adhesive layers, and the assembly was placed on top of the conductive layer. The adhesive was trimmed to match the conductive layer’s size, and the protective layer was removed to expose the other side of the adhesive. The outer layer was wrapped around a finger and then rolled onto the adhesive to prevent air pockets. This process was repeated for the other side of the conductive layer with the transverse wires. The longitudinal wires were soldered and encapsulated in heat shrink tubing separately, while all the wiring along the fingers was encapsulated together in heat-shrink tubing together (Electriduct, 3.18 mm 3:1 Polyolefin Tubing). The soldering of the horizontal channels was performed after the Dragon Skin-30 cured to prevent any unnecessary strain on the steel thread during fabrication. The finished size of the sensor was 1.5 cm × 1 cm.

### 2.2 Designing the molds to cast the soft exoskeleton

SolidWorks 2019 software was utilized to create molds for the hand exoskeleton, taking into account measurements obtained from prior anthropometric studies (Garrett, 1971). A second mold was produced that allowed for an additional 0.3 cm of width and 0.4 cm of thickness to accommodate the wiring of the sensor array; slots were included for the wire conduits. To ensure proper placement of the actuators, semi-cylindrical rods with a 0.35 cm cavity at one end were designed and inserted into the molds. Caps were printed to cover the openings of the fingers in the first mold, featuring an opening for the rods to maintain their alignment. Both molds and caps were printed using the Ultimaker S5 (Ultimaker, Netherlands) and made of PLA (Overture, PLA, 2.85 mm filament). The rods acted as placeholders for the air channels and were 3D printed using the Ultimaker S5 and dissolvable PVA (Polymaker, PolyDissolve S1, 2.85 mm filament).

### 2.3 Fabricating the hand exoskeleton

**FIGURE 2:**
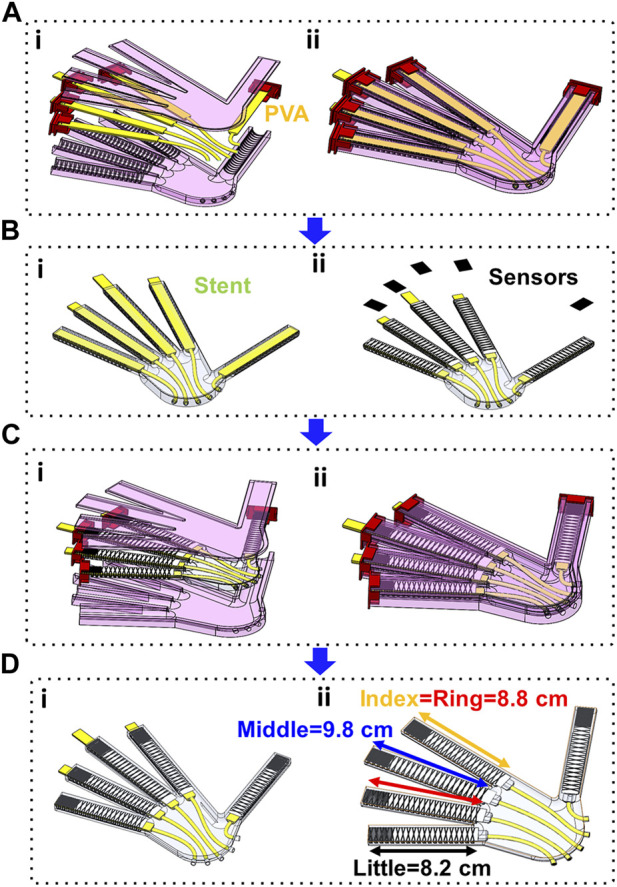
Manufacturing Process: **(A)** (i) All printed components in CAD assembly, cast made from mold 1, PVA stents, and tubing, (ii) Complete stage 1 cast, shown after filling with Dragon Skin and sealing shut; **(B)** (i) Result of stage 1 cast, (ii) Cast is equipped with strain-limiting layers and pressure sensor arrays; **(C)** (i) Fully equipped cast is lain in the stage 2 mold to encase the strain limiting layers and sensors as part of the exoskeleton, (ii) Complete stage 2 cast, shown after filling with Dragon Skin and sealing shut; **(D)** (i) Result of the stage 2 cast, (ii) PVA stents are dissolved, and stage 3 casting is done to seal the pressure chambers.

The process began by inserting polyurethane tubing (1.59 mm × 3.18 mm) through the mold’s holes and then into the stents (Figure 2A). To make it easier to remove later, the stents were wrapped with Teflon (Sklety, PTFE Pipe Sealant Tape). The stents were positioned inside the caps such that their bottom edge aligned with the base of each finger and ran parallel to the molding. Hot glue was also used to temporarily secure the tubing and stents in place as required. The mold was then filled with 120 g of hydrogel material (Smooth-On, Dragon Skin-30) and allowed to be cured for 4 h before cleaning any excess material. Before molding the outer layer of Dragon Skin, each finger had a rectangular piece of fabric (100% Blackout Grommet Banton Window Curtain Panel, S.L. Home Fashions Inc.) placed over the flat base for reinforcement. Carbon fiber tow (1K 3800 MPa 50/100 m Length Carbon Fiber Fibre Tow Filament Yarn Thread Tape, AliExpress) was then wrapped in a helical pattern around the silicone cast. The fiber was wrapped first from the base of the finger to the tip and then from the tip to the base, intersecting itself at the apex of the dorsal side and along the palmar side (Figure 2B (ii)).

The end of each finger had the tactile sensor arrays installed with all vertical wires already soldered in place. To provide greater mobility during pre-mold placement and actuation, the wires for each sensor were separated into two bundles (transverse and longitudinal) and covered with heat shrink. Each tactile sensor comprised eight wires that ran through the palm to a 40-pin cable connector (Micro SATA wiring, IDE cable). The tubing and wiring were placed in the slots in the mold and filled with 80 g of Dragon Skin, then left to cure for 4 hours (Figure 2C). Once the curing was done, the horizontal wires of the sensors were soldered to the corresponding wires of the 40-pin cable connector.

In stage 3 of the molding process the excess rubber around each finger was trimmed to a uniform length of 3 mm from the sensor array’s edge (Figure 2D). The PVA stents were dissolved in water and the Teflon was removed from the inside surface. Next, the end of each finger was dipped in a cup filled with 10 g of Dragon Skin, and after a 4-h curing period, the excess material was removed to leave a Dragon Skin cap that completely sealed the pneumatic chambers. The palm side of the hand containing the tubing was cut open, leaving a layer of silicone rubber around the tubing, and a zip tie (Outus Nylon Cable Ties) was fastened around the Dragon Skin at the inlet of each finger actuator to ensure an airtight seal. Any gaps were filled with Dragon Skin using an open molding process.

### 2.4 Soft actuator characterization

To evaluate the force response and hysteresis of the soft actuators, three internal pressures were used for testing. The force of the fingertips (little, ring and middle finger) was measured individually using a 2 kg load cell (LSP-2, Transducer Techniques, Temecula, United States), and each test comprised 16 cycles, with 3 s of actuation followed by 7 s of rest. The 16 cycles were recorded three times at pressures of 0.14, 0.21, and 0.28 MPa. The forces and pressures obtained from these tests were used to plot the hysteresis at each of the three pressures. The maximal force at pressures of 0.14, 0.21, and 0.28 MPa was obtained from the force-pressure relationship for each finger.

### 2.5 System configuration for perception and action

**FIGURE 3:**
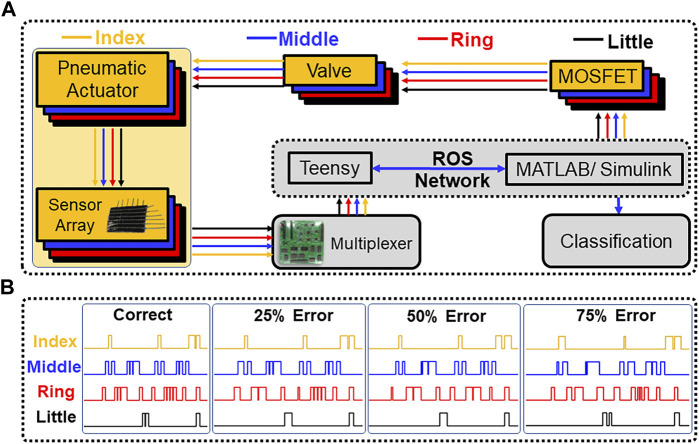
Control system. **(A)** The control scheme for the exoskeleton and sensors; **(B)** The valve control signals of each finger for playing “Mary Had A Little Lamb.” Illustrative examples are shown of the correct song and the song variations that had errors introduced.

A Teensy 4.0 microcontroller and a multiplexer board were used to sample the 16 taxels on each of the fingertips. The resistance of each taxel was used in a non-inverting op-amp circuit and the output voltage of the op-amp circuit was measured for each of the 16 taxels on every digit of the exoskeleton at 74 Hz. To cycle through the 80 available taxels, the multiplexer (Sundaram et al., 2019) was used, and the output voltage from the op-amp circuit was sampled ten times per taxel and averaged by the Teensy. The data was then published to a Robot Operating System (ROS) network from the Teensy, and Simulink was linked to the same ROS network for real-time data visualization and storage (Figure 3A).

Simulink was used to implement the 10 song variations by controlling the valves of the four fingers. The control inputs for the four valves were created with the Simulink signal builder block and set as four separate digital outputs. These outputs were then used as a 1 V input to a MOSFET (NTE2389), which controlled the 24 V signals for the 5 Festo solenoid valves (MHE2-MS1H-3) that were responsible for controlling the fingers. A single pressure reservoir at 0.28 MPa was connected to all five valves, and the open/closed state of the valves was determined by the 1 V outputs from Simulink, which controlled the pressure applied within the pneumatic actuators.

### 2.6 Exoskeleton to (Re)Learn playing the piano

To assess the potential of using the smart hand exoskeleton for rehabilitation purposes, we programmed it to play ten different versions of the well-known tune “Mary Had a Little Lamb.” To introduce variations in the performance, we created a pool of 12 different types of errors that could occur at the beginning or end of a note, or due to timing errors that were either premature or delayed, and that persisted for 0.1, 0.2, or 0.3 s. By combining these error types, we generated 12 unique error scenarios that were included in the different song variations. We created three groups of three songs each based on the number of errors present: the first group had 75% of the keys played with some type of error, the second group had 50%, and the third group had 25%. Within each group, we created three variations, each with errors on the same note but with the error type randomly selected from the pool of options. The ten different song variations consisted of the three groups of three variations each, plus the correct song played with no errors (Figure 3B).

The study included 20 repetitions of each of the ten song variations in two settings: with the hand exoskeleton worn by a human subject (25-year-old male, able-bodied) and with the exoskeleton playing the songs independently (without a person). The study was conducted with institutional review board oversight and the human subject provided written informed consent in accordance with the Declaration of Helsinki. The resulting datasets from the 200 song repetitions played were used to train three machine learning classification algorithms (KNN, RF, ANN), which were then evaluated based on their ability to distinguish between the different song variations. This approach could potentially provide real-time feedback to individuals recovering from a stroke or other neurotrauma who are (re)learning to play a musical instrument.

### 2.7 Machine learning classification methods

To design the machine learning problem, 10 different song alterations were programmed for the hand to perform (Figure 3B). For each song alteration, 20 repetitions were collected to perform machine-learning tasks. The collected data were preprocessed and labeled to train the RF, KNN, and ANN algorithms to separate the data into 10 different classes corresponding to the 10 song variations. The KNN algorithm was used to calculate the shortest distance between a query and all the points in the features and select the specified *k* number closest to the query and vote for the most frequent class label (Zhang et al., 2017a). The RF algorithm resembled a tree structure that can be trained separately to perform the classification (Breiman, 2001). The decision trees were the main components of this algorithm. In general, the more trees in the forest the more robust the prediction which leads to higher reported accuracy. The last algorithm was the ANN (Basu et al., 2010) which was trained and evaluated using cross-entropy and confusion matrices. A two-layer feed forward network with sigmoid hidden and softmax output neurons was used to classify the collected data into ten classes for the different song alteration classes. The network was trained with scaled conjugate gradient back propagation.

To train the KNN and the RF classifiers, the collected datasets were divided into two sets: the training dataset, which contains 80% of the data, and the testing dataset, which contains 20% of the collected data. However, to train and test the ANN, the collected data were divided into 3 categories: 70% for training, 15% for validation, and 15% for testing. The training dataset was presented to the network during training, and the network was adjusted according to its error. The validation dataset was used to measure network generalization and to halt training when generalization stopped improving. The testing dataset did not affect training and so provided an independent measure of network performance during and after training. Training automatically stopped when generalization stopped improving, as indicated by an increase in the cross-entropy error of the validation samples.

To verify the accuracy of each algorithm, they were run ten times for each song variation with a randomized selection of the training and testing data. The mean and standard deviation of the classification accuracy were calculated for each algorithm. A two-factor analysis of variance (ANOVA) was performed. The first independent variable was the classification algorithm (RF, KNN, ANN). The second independent variable was whether the hand exoskeleton was worn by a person or used independently. The classification accuracy of the machine learning algorithms was the dependent variable. A *p*-value of 0.01 was assumed for statistical significance.

## 3 Results

### 3.1 Performance of soft actuators

**FIGURE 4:**
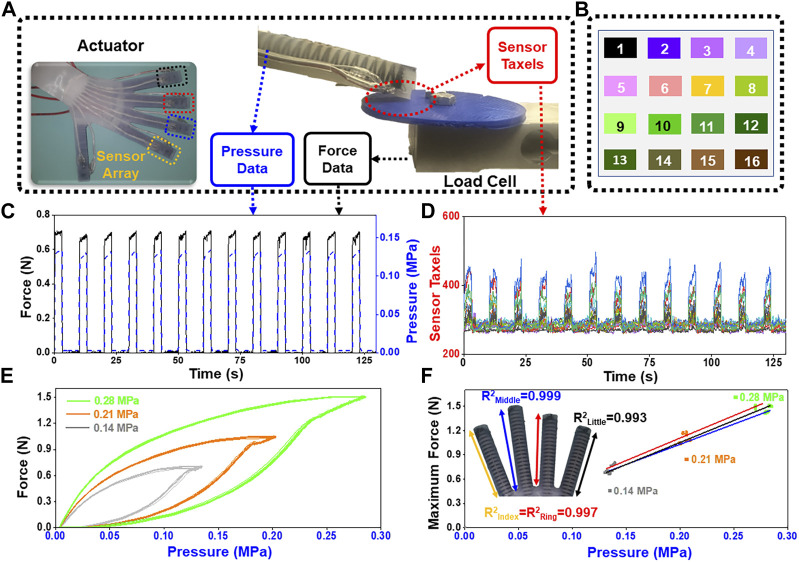
**(A)** The hand exoskeleton was equipped with an internal pressure sensor and applied forces to the load cell; **(B)** Color map shows the spatial location of the 16 taxels on the sensor of each finger; **(C)** Force measured by the load cell for the little finger at an internal pressure of 0.14 MPa; **(D)** Corresponding taxel signals at the pressure of 0.14 MPa; **(E)** Force-pressure relationship using 16 actuation cycles of the little finger for three different internal pressures; **(F)** the maximal generated fingertip forces correlated almost linearly to increasing pressure over the tested range.

The response of each soft actuator was highly repeatable with each of the three applied pressures (Figure 4). With an internal pressure of 0.14 MPa, the average maximum fingertip force and standard deviation were 0.72 ± 0.04 N. With an internal pressure of 0.21 MPa, the mean and standard deviation were 1.10 ± 0.05 N, and at 0.28 MPa pressure, the mean and standard deviation were 1.47 ± 0.04 N (Figures 4A, C, E). Illustrative data of the sixteen taxels in a fingertip (Figure 4D) show the response of the tactile sensor when the finger was internally pressurized to repetitively apply forces to the load cell (Figure 4D). The hysteresis of the actuators follows a characteristic trend with each internal pressure (Figure 4E). The maximal generated fingertip forces had a near linear correlation to increasing pressure over the tested range (Figure 4F). A linear model that was fit to these data had an *R* ^2^ value of 0.993 for the little finger, 0.997 for the ring finger, and 0.999 for the middle finger.

### 3.2 Feeling the beat: classification accuracy for piano playing

**FIGURE 5:**
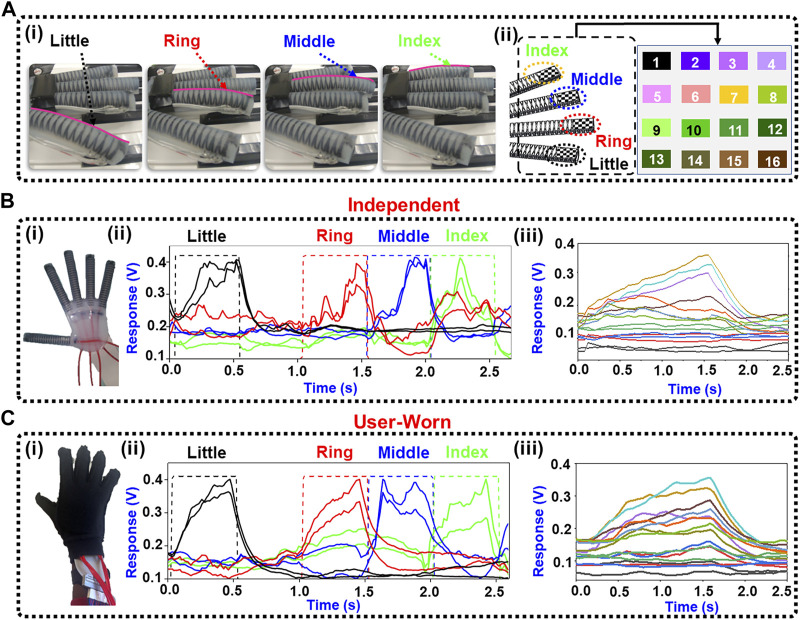
Exoskeleton playing the piano independently and while being worn. **(A)** (i) Actuation of each finger while playing a song. (ii) Color map of the tactile sensor showing the locations of taxels on each finger; **(B)** (i) The exoskeleton as used independently, (ii) Two illustrative taxels for each finger are shown while playing the song. (iii) The response of all the taxels for the little finger during a single keystroke; **(C)** (i) The exoskeleton was inserted into a glove and worn by the human subject. (ii) Two illustrative taxels for each finger. (iii) The response of all the taxels for the little finger from a single keystroke.

The soft robotic hand exoskeleton played the 10 song variations independently and while being worn by a user. Figure 5A(i) displays the hand operating independently to press the keyboard. The independent data are shown first in Figure 5B, followed by the user-worn data for comparison (Figure 5C). Figure 5B(ii) and Figure 5C(ii) show two illustrative taxels from each finger for clarity. The normalized response of all 16 taxels from the little finger in a single keystroke is shown in Figure 5B(iii) and Figure 5C(iii) for the independent and user-worn situations, respectively.

**FIGURE 6:**
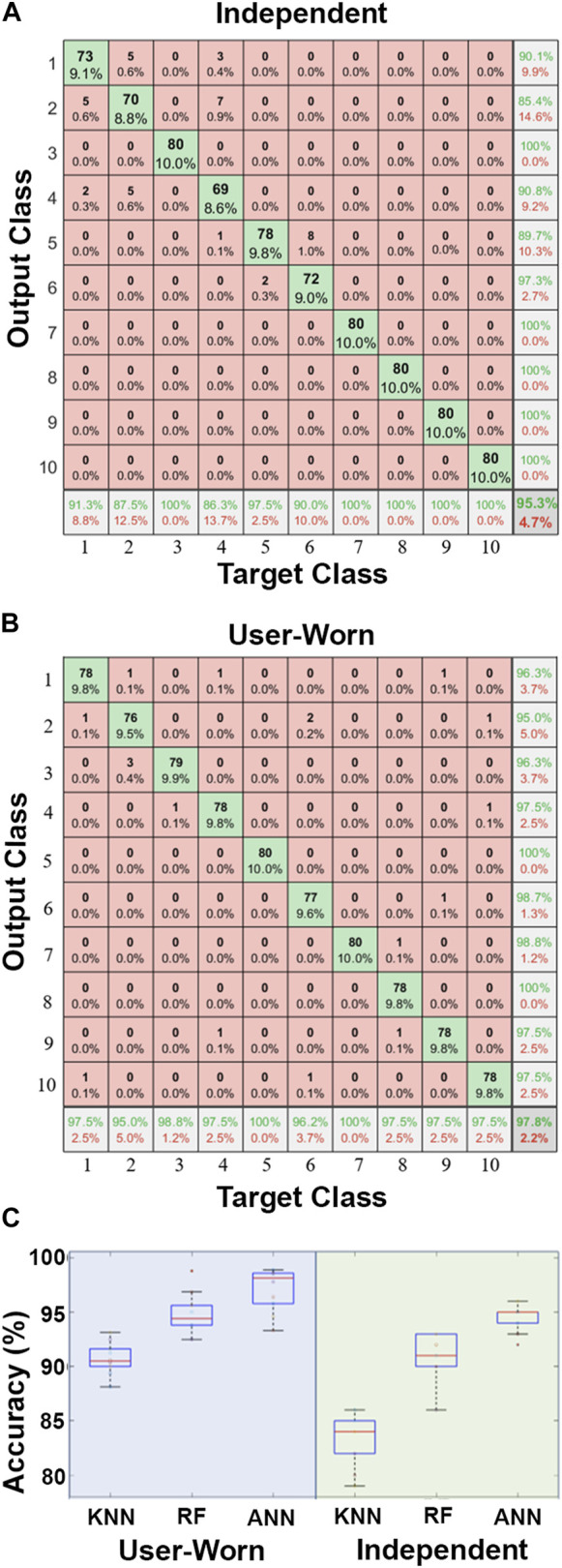
**(A)** Illustrative confusion matrices for the ANN showed the accuracy for classifying the 10 different song alterations during independent use and **(B)** while user-worn; **(C)** Comparison of 3 classification algorithms during independent use and with a human subject wearing the soft robotic exoskeleton. The ANN had significantly higher accuracy than the KNN and RF algorithms.

The ANN achieved the highest classification accuracy with 94.60% ± 1.26% for independent use and 97.13% ± 2.00% when worn by a person (Figures 6A, B). The RF had a classification accuracy of 91.00% ± 2.11% for independent use and 94.77% ± 1.96% when worn, while the KNN had the lowest classification accuracy with 83.30% ± 2.45% independently and 90.70% ± 1.48% when worn (Figure 6C). Results from the two-factor ANOVA indicated that the classification algorithms were significantly different from each other (*p* < 0.01). There was a statistically significant difference in accuracy between independent and worn usage (*p* < 0.01), and there was also an interaction effect between the two independent variables (*p* < 0.01).

## 4 Discussion

We designed a novel soft exoskeleton using 3D printed PVA stents and hydrogel casting to integrate 5 actuators into a single wearable device that conforms to the user’s hand. The fabrication process is new, and the form factor could be customized to the unique anatomy of individual subjects with use of 3D scanning technology or CT scans. We also developed a flexible tactile sensor array that was embedded into each fingertip of the exoskeleton, with each array containing 16 taxels. To our knowledge, all these features have yet to be combined into a single hand exoskeleton. We used artificial intelligence to ‘feel’ the difference between correct and incorrect versions of a song played on the piano as one illustrative demonstration of the potential for the smart exoskeleton to be used as a rehabilitation tool to improve hand dexterity.

Our approach to designing the hand exoskeleton involved using 3D printing techniques to create a complete soft robot that could be based on the patient’s needs. Zhao et al. (2016) incorporated a 3D-printed rigid palm and wrist into a fiber-reinforced soft prosthetic hand. Our complete soft palm and wrist design approach could further improve the effectiveness of their application. Furthermore, the use of 3D-printed PVA stents in our new soft robotic hand provided an added advantage. Compared to the fabric-based soft robotic glove developed by Cappello et al. (2018) for individuals with upper limb paralysis following spinal cord injury, our approach involved fewer fabricated layers, making the process less complicated. The PVA stents can be easily dissolved in water to aid in the removal of Teflon from the inside surface. Additionally, we embedded the flexible sensor array during the fabrication process to allow for tactile sensations, which could be readily adjusted based on the user’s dimensions. Fras and Althoefer (2018) proposed a soft pneumatic hand capable of passively adapting to grasped objects due to its mechanical compliance. The inclusion of sensors within a design such as this would aid in the control of the device.

In the past, other soft robotic actuators have been used to play the piano; however, ours is the only one that has demonstrated the capability to ‘feel’ the difference between correct and incorrect versions of the same song. Takahashi et al. (2020) developed a soft hand exoskeleton that allowed pianists to move their fingers more easily and quickly, without imposing as many limitations as possible on their voluntary movements. The actuator also reduced variability in the force of the keypresses even when the pianists were not wearing gloves. However, our soft robotic hand actuator utilized machine learning trained by tactile sensor arrays that could be used to provide instructive feedback to users and useful data for clinicians. This makes it an ideal tool to assist disabled individuals in relearning how to play the piano correctly. This unique capability is enabled by the novel design at the intersection of flexible tactile sensors, soft actuators, and artificial intelligence that would be handy when applied to many other tasks beyond musical instruments. Hoang et al. (2021) utilized a random access pneumatic memory device to regulate the soft robotic fingers that play the piano. This approach helped to reduce the amount of hardware required to control multiple independent actuators in pneumatic soft robots. Wang et al. (2022) developed and produced a three-fingered soft-rigid hybrid hand system with variable stiffness in each finger. Their design enabled diverse compliant behaviors for pressing piano keys. In comparison, our smart hand exoskeleton can expand their range of applications by incorporating sensing technology and artificial intelligence to characterize the interaction between the environment and the robot.

The three classification algorithms we employed in this paper had significantly different accuracies. The classification performance of various algorithms can differ depending on the task, dataset, and available resources. In this study, the classification accuracy of the ANN surpassed that of the KNN and RF for several reasons. First, ANNs possess many internal parameters (weights and biases) within interconnected neurons and layers, granting them the flexibility to fit highly complex datasets with superior classification accuracy compared to other models like KNN and RF, which may achieve lower accuracy in such cases (Breiman, 2001; Zhang et al., 2017b). Additionally, ANNs can automatically learn intricate feature representations from raw datasets (Bergen et al., 2019). By learning hierarchical representations through multiple layers, ANNs can capture complex patterns in the data, whereas KNN and RF rely on simpler distance metrics that may struggle to capture such complexities. ANNs excel in learning nonlinear relationships between input features and output predictions, enabling them to model intricate decision boundaries and capture more intricate patterns. However, it is worth noting that the performance of these algorithms depends on the specific task, dataset characteristics, and hyperparameter tuning. In certain cases, KNN or RF may be more suitable or outperform ANN, particularly for small datasets or where interpretability is prioritized. Careful consideration of the trade-offs and characteristics of each algorithm is crucial when selecting the most appropriate one for a given problem.

All three classification algorithms had higher accuracy when the exoskeleton was worn compared to when used independently (Figure 6C). This could potentially be caused by better pressure distribution across the surface of the tactile sensor when it was worn. When used independently, the rigid piano key made direct contact with the taxels near the sensor tip, which inherently causes pressure concentrations in localized areas. In contrast, when the sensor was in contact with a human hand, the higher compliance was more likely to distribute the pressure more evenly across the surface of the tactile sensor to consistently activate more taxels. These likely created a greater difference between activation and rest states that was more recognizable by the machine learning algorithms when the exoskeleton was worn by a person.

The successful detection of song errors can provide measurable outcomes for patients in their rehabilitation programs. Although this study’s application was for playing a song, the approach could be applied to myriad tasks of daily life. Thus, the device could facilitate intricate rehabilitation programs customized for each patient. The current machine learning algorithm can successfully determine the percentage error of a certain song as well as identify key presses that are out of time. Clinicians could use the data to develop personalized action plans to pinpoint patient weaknesses, which may present themselves as sections of the song that are consistently played erroneously and can be used to determine which motor functions require improvement. As patients progress, more challenging songs could be prescribed by the rehabilitation team in a game-like progression to provide a customizable path to improvement.

Furthermore, the rehabilitation approach using smart exoskeletons demonstrated in this study can be extended to a wide range of injuries particularly if used in conjunction with other devices designed for the elbow and shoulder (O’Neill et al., 2017; Nassour et al., 2021). Additionally, a larger tactile sensor could readily provide functionality for assessing progress towards achieving a normal force pattern for shoulder and elbow exoskeletons. Soft actuators offer the advantages of conformability, ergonomics, and design versatility through 3D printing and casting methods. Corresponding flexible sensing technologies are essential for the use of soft exoskeleton technologies. The development and implementation of new sensor arrays within these exoskeletons will significantly expand their range of applications and improve their effectiveness.

The effect of many different users’ biomechanics on the soft exoskeleton’s response was not investigated in this paper, though it will be added as an avenue of future investigation. In future works, we have also considered numerous potential ways to convey training feedback to the user. These include visual feedback with a smartphone (Yin et al., 2021) and haptic feedback with a wearable device like a smartwatch (Heng et al., 2022). We have also considered adding vibrotactile feedback within future versions of the smart exoskeleton that could vibrate to alert the users of errors (Pan et al., 2018).

## 5 Conclusion

A new soft exoskeleton was designed with integrated tactile sensor arrays that were used for the novel application of ‘feeling’ the differences between correct and incorrect versions of a song played on the piano. The RF, KNN, and ANN algorithms were trained by data from the 80 taxels in the four fingertip tactile sensors that each had 16 taxels. The ANN algorithm achieved the highest level of accuracy, attaining a success rate of 97.13% ± 2.00% when tested with a human subject, and 94.60% ± 1.26% while used independently. Furthermore, we introduced a new hand exoskeleton fabrication technique that used 3D printed PVA stents and hydrogel casting to incorporate 5 actuators into a complete wearable device. The smart soft exoskeleton demonstrated high classification accuracy and could be used in the future to guide disabled people on fully customized rehabilitation programs.

## Data Availability

The original contributions presented in the study are included in the article/Supplementary Material, further inquiries can be directed to the corresponding author.
