# Supplementary material for: Feeling the beat: a smart hand exoskeleton for learning to play musical instruments
Source: Front Robot AI. 2023 Jun 29;10:1212768. doi: 10.3389/frobt.2023.1212768 (PMC10338871; doi:10.3389/frobt.2023.1212768)
Supplement: Supplementary file 2 [file DataSheet1.docx]

Fig. S1. The response of all the taxels for the little finger during a single keystroke. A. A single note played during the correct song and B. incorrect song variations when the exoskeleton was used independently. C. A single note played during the correct song and D. incorrect song variations when the exoskeleton was worn by the human subject.
